# Interview with a Retrovirologist: Wibke Bayer in conversation with Hung Fan

**DOI:** 10.1186/s12977-022-00597-1

**Published:** 2022-07-04

**Authors:** Hung Fan, Wibke Bayer

**Affiliations:** 1 Department of Molecular Biology & Biochemistry, and Cancer Research Institute, University of California, Irvine, CA 92697 USA; 2 Institute for Virology, University Hospital Essen, University Duisburg-Essen, 45122 Essen, Germany

*“Retrovirology is pleased to share the next installment in “Interview with a Retrovirologist”, in which two scientists discuss their careers, with the goal of highlighting leaders and rising stars, celebrating diversity and inspiring the next generation of scientists. Our latest pair of scientists is Dr. Hung Fan of the University of California, Irvine, and Dr. Wibke Bayer, from the University Duisburg-Essen. We learned a lot about both Hung and Wibke from this piece, and hope that the readers of Retrovirology find it equally thought-provoking”*.

## Hung Fan: biography

**Fig. 1:**
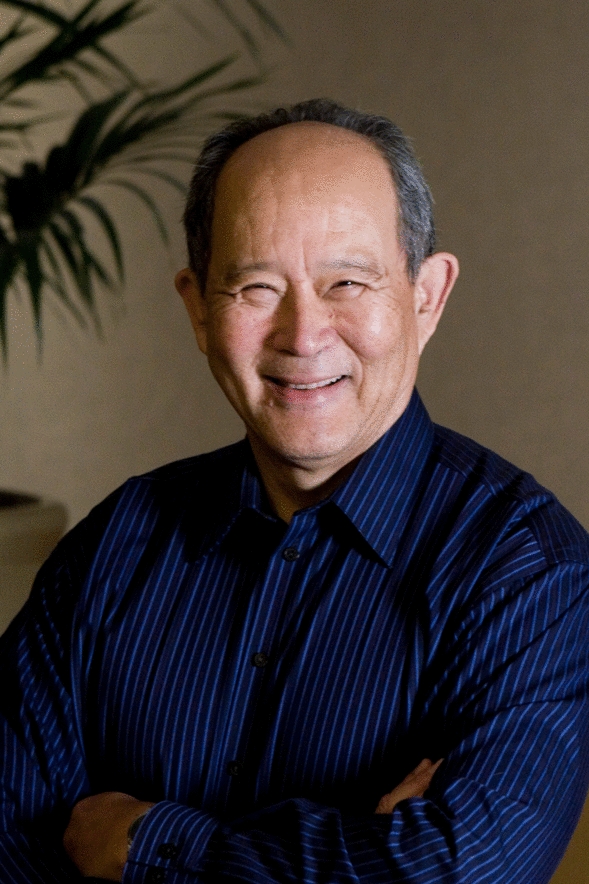
Hung Fan bio photo

Hung Fan (Fig. 1) was born in Beijing, China and immigrated to the US with his family when he was one year old. He grew up in West Lafayette, Indiana where his father was on the faculty of Purdue University. He did his undergraduate studies in physics at Purdue, and he was a graduate student in biology at MIT, where he received the Ph.D. in 1971. His graduate work was under Sheldon Penman, a pioneer in mammalian cell molecular biology; his thesis was on RNA and protein synthesis in metaphase-arrested cells. He was a postdoctoral fellow with David Baltimore, also at MIT, where he began studies on viral RNA in murine leukemia virus-infected cells. Fan began his independent research career in 1973 at the Salk Institute where he continued his studies on MuLV. He moved to the University of California, Irvine in 1981 where he rose through the ranks to full professor, retiring in 2015.

His studies on MuLV include characterizing synthesis and processing of viral RNA, molecular cloning of the Moloney MuLV genome, characterizing enhancer sequences in the viral long terminal repeat (LTR), and the multi-step process of leukemogenesis. In 1978 he discovered MuLV glycosylated Gag (glyco-Gag), a protein that is now understood to counteract cellular resistance factors including APOBEC3 and SERINC5. His lab also obtained an infectious molecular clone of jaagsiekte sheep retrovirus (JSRV), the etiologic agent of a transmissible lung cancer in sheep. Noteworthy findings were that the envelope protein is also an oncogene, the viral LTR is transcriptionally specific for lung epithelial cells, and JSRV encodes a rev-like regulatory protein. He has published over 200 papers and reviews. Fan was director of the UCI Cancer Research Institute for 30 years, and Co-Director of the Chao Family Comprehensive Cancer Center. He has organized more than 60 scientific meetings, including international meetings on retroviral pathogenesis, the Palm Springs symposia on HIV/AIDS, and the West Coast Retrovirus meetings. Fan is an elected fellow of the American Association for the Advancement of Science, and the American Academy of Microbiology, and he has given several named lectures. He continues at UCI as Associate Vice Chancellor for Strategic Initiatives.

## Wibke Bayer: biography

**Fig. 2:**
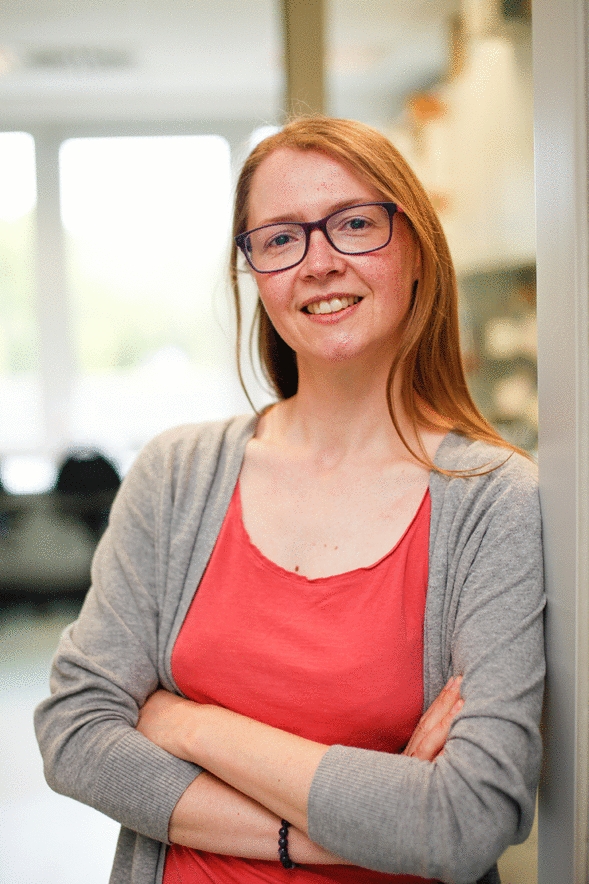
Wibke Bayer bio photo

Dr. Wibke Bayer (Fig. 2) is a group leader at the Institute for Virology at the University Hospital Essen of the University Duisburg-Essen in Germany. She studied Biochemistry at the Ruhr-University in Bochum, Germany, and completed her diploma and doctoral theses in the Department of Molecular and Medical Virology under the mentorship of Dr. Oliver Wildner. After joining Prof. Ulf Dittmer’s lab at the University Hospital Essen in 2010, she established her research group that focuses on the development of adenovirus-based vaccine vectors for immunization against retrovirus infections and on the pathogenesis of the Friend retrovirus infection of mice, with a current focus on the immunosuppression by retrovirus Env.

*WB: The most basic and most obvious question first, what made you become a retrovirologist? I believe I know that you majored in physics so it was kind of a leap to go into molecular biology I suppose, and into molecular oncology?*

HF: OK, I was born in mainland China, and my family immigrated to the US in 1949 when I was a year and a half old. My father was a physicist at Purdue University, and that's where I grew up. I had an older brother who in many ways set an example. Like him, I went to Purdue for my undergraduate degree and majored in physics. I always had a basic interest in biology when growing up, but studying physics was a good background for modern biology, and also at the same time I did complete almost all of the requirements for a major in biology. For my graduate studies, I applied to several schools, and MIT was one of them. Even though I did not intend to follow in my brother’s steps, who had also obtained his Ph.D. in biology there five years before, I still felt it was the best fit for me. I did my Ph.D. with Sheldon Penman who was one of the pioneers in mammalian cell molecular biology. For my Ph.D. thesis, I studied RNA and protein synthesis during metaphase, either in HeLa or Chinese hamster ovary cells. While I was trained as a molecular cell biologist, at the same time the general topic of virology really resonated with me. I just was intrigued with it, and one of the yardsticks was that I could remember trivial facts in virology if I read them once. In other areas of biology, I could read papers several times and they wouldn't stick—for example immunology which at that time was not the molecular science that it is now. Many immunological principles were defined by indirect experiments, and that did not resonate with me.

While I was a graduate student at MIT, I took a course on animal virology with a young professor who also interacted a lot with graduate students and postdocs, David Baltimore. I actually heard about his discovery of reverse transcriptase in 1970 before it was published. His teaching really shaped my approach to virology—he had a beautiful way to think about viruses in general. When discussing any different virus, he always approached it in a structured, systematic way: what is the structure of the virus, what are its contents, how does it replicate? It was fascinating to hear him talk about viruses during this time. When I was getting toward the end of my Ph.D. (and right after his discovery of reverse transcriptase), I asked David if I could post doc with him. My intent was to use new tools that were now available, in particular reverse transcriptase, to study the molecular biology of RNA tumor viruses, now known as retroviruses. He said yes, and that's how I got into retrovirology.

For the younger people who are reading this, the reason I was so excited about joining David's lab and working on the RNA metabolism of murine leukemia virus was, this was at a time before molecular cloning. In the absence of molecular cloning, how could you make a hybridization probe to study a gene of interest? Virtually all early molecular studies of gene expression in mammalian cells used viruses. For DNA viruses, you could make hybridization probes by isolating virion DNA and radioactively labeling it by nick translation. With the discovery of reverse transcriptase, it became clear that you could make hybridization probes for RNA tumor viruses, by incubating purified virus particles in an endogenous reverse transcriptase reaction in the presence of radioactive DNA precursors. Then it would be possible to study the RNA metabolism of those viruses. That was the first stage in my career.

*WB: How did you then transition into a PI position?*

HF: I postdocced with David at MIT after completing my Ph.D. for about two years, studying the RNA metabolism of murine leukemia virus in infected cells. During that time, David was invited to spend a summer at the Salk Institute, where he had his first independent research position, loosely allied with Renato Dulbecco in the Tumor Virology Lab. Dulbecco was moving to England to the Imperial Cancer Research Fund labs. The Salk was looking to replace Dulbecco and fill his lab, and they invited David to come and bring four of his postdocs for the summer and I was one of them. We spent 6 weeks doing experiments and enjoying the La Jolla lifestyle. David ultimately declined the position at the Salk since he was committed to the MIT Cancer Center. And so the Salk changed direction and decided that instead of hiring a big gun, to hire some junior faculty. They asked David for recommendations, and I was one of those he recommended. I took the job at the Salk Institute, along with other new, young investigators including Inder Verma, Bart Sefton, Tony Hunter, Rudolph Jaenisch and Gernoth Walter.

That was the path that took me to retroviruses, both scientifically as well as physically if you will.

*WB: Would you say it was more important that David Baltimore himself was very inspiring and a very approachable group leader? Or was it more the techniques and new technical possibilities that attracted you?*

**Fig. 3:**
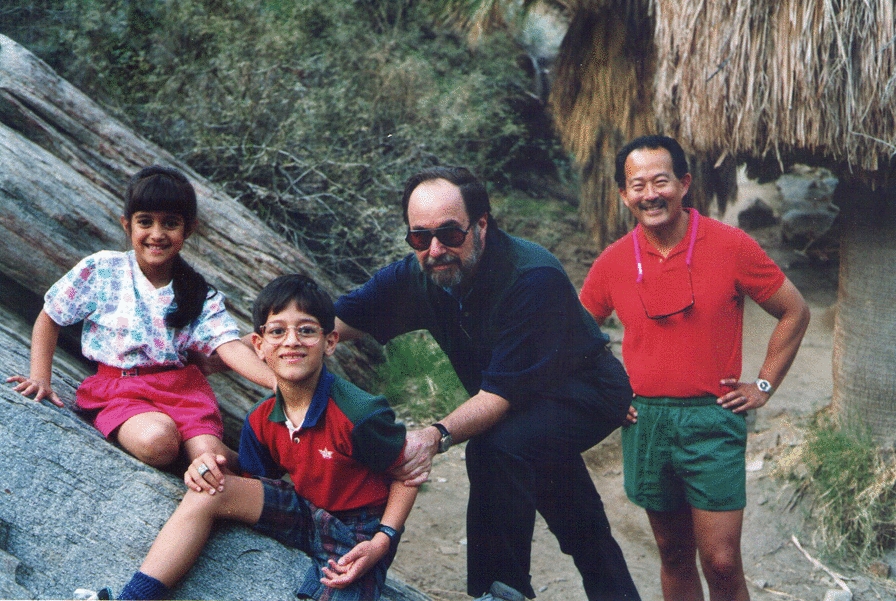
1995—a walk in Palm Canyon with David Baltimore (and two of Ramesh Akkina's kids) during a Palm Spring Symposium on HIV/AIDS

HF: It was both. I like to contrast my two advisors, my Ph.D. advisor, Sheldon Penman, and David Baltimore, my postdoctoral advisor. Penman was someone who interacted with his graduate students on a daily basis, and would really get into the weeds of looking at results. Even if they were rather lousy looking data, with some obvious flaws, he was perfectly happy to look at it and see what you could learn. That was something that I very much benefitted from. One time I had worked very hard on a challenging series of experiments that we ultimately published in the Journal of Molecular Biology. It took many tries to make it work perfectly, and when I finally had got a really nice demonstration, I brought it to him and he said “Ah, you have finally done the typical experiment!” In those days, papers often had phrasing like “*A typical experiment is shown in Fig. 8*”. Sheldon also really taught us how to strategically approach scientific problems. In later years, if I met others who had trained with him, if we were talking science, we would often think of the same experiments to do. On the other hand, David was a great post-doctoral advisor for me (Fig. 3). I already came with the technical capabilities for experimentation and approaches I learned from Sheldon. Where David really was great was when you had the framework of a story—you already had the key data and pretty much knew what the story was. He really could help to shape it in terms of thinking strategically about it and its implications, and where you should go from there.

*WB: When you set up your lab at the Salk Institute, you were with a group of rather young group leaders, so that must also have been a quite dynamic environment then, with all the people just getting new things started, in contrast maybe to a very established lab?*

HF: Yes, definitely. Sometimes, institutes or large departments can also be very hierarchical, where there’s the big leader or professor and everybody is working either in their shadow or along the tracks that are already laid down. This new Tumor Virology Lab was a great place to start a career because you had people who were working on different systems but who were at the same professional level. There was a lot of both enthusiasm and support for each other, both collaboration-wise as well as technically, and also emotionally.

*WB: At the time, it was called tumor virology lab. Did you think of yourself as a retrovirologist? Or did you consider yourself a molecular oncologist, since you were very involved in cancer research? Also later, your job title was Professor for Molecular Biology.*

HF: I think I almost always thought of myself as a virologist. The questions that I was interested in really turned around virology and ultimately how viruses cause disease. A number of my other colleagues in the TVL went in different directions. Tony Hunter did groundbreaking work in retroviruses, particularly Rous sarcoma virus and how the *src* gene works. Inder Verma did a lot of work in very important parts of retrovirology, first with the biochemistry of reverse transcriptase. They ultimately went in different directions into cell biology, cancer biology, and so forth. I've pretty much stayed with the virological aspects in my career.

*WB: Yes, you have done a lot of work on Moloney murine leukemia virus. So what made you focus on this particular virus?*

HF: I cannot say that the reason I started working on Moloney virus was anything inspired. I knew I wanted to study the RNA metabolism of an RNA tumor virus using reverse transcriptase. The original experiments that Baltimore did were on Rauscher murine leukemia virus, and the reason was that he obtained relatively large amounts of it from the National Cancer Institute. At that time, the two big RNA tumor viruses were Rous sarcoma virus, and the murine leukemia viruses. Howard Temin (along with Peter Vogt and Peter Duesberg) was a major leader in Rous sarcoma virus. Mostly, people tended to use Rous sarcoma virus in short-term experiments, to study the ability of a virus to transform cells and change their phenotype, to turn them into tumor cells. Murine leukemia viruses on the other hand did not actually do that, but they were more amenable to carrying as long-term infected cultures that continually liberate virus. Such a culture was the source of the purified Rauscher murine leukemia virus that was used by Baltimore.

After I joined the lab and I needed productively infected cells for my studies, we asked for the producer cells. The NCI contractors who produced the virus told us that they didn’t have the Rauscher producer cells in culture right then, but they had a related cell line in culture that produced Moloney virus. We said, okay, we’ll take the Moloney virus producers. And that’s the reason why essentially all of the molecular biology work coming out of the Baltimore lab was Moloney virus based as opposed to let’s say Rauscher or Friend virus. I was the first person in the Baltimore lab to culture, biologically clone and purify Moloney virus.

*WB: Looking back, what would you say was the most important finding that you made? And maybe, on the other hand, if it's a different answer, what did feel most rewarding to you when you made the discovery?*

HF: Well, that’s a good question. I think some of our important early work was looking at the viral RNA in infected cells. At that time, what we now take for granted was not known, and a lot of what we did established that. The first question was, what is the size of virus-specific RNA in infected cells? We determined that the size of virus-specific RNA in infected cells is the same size as the genomic RNA, as well as subgenomic RNA. Another question was, what is the size of viral messenger RNA? We determined that there is viral messenger RNA that is genome-length, as well as viral mRNA that is smaller (subgenomic). We addressed how to associate a particular mRNA with the protein that’s being synthesized. For that, we prepared a rabbit polyclonal antibody against the viral capsid–we still get asked for it since it’s a highly specific, high-affinity antibody. We used the CA antibody to immunoprecipitate polyribosomes form virus infected cells and we found that the viral mRNA that encodes the Gag protein is the full-length mRNA.

Another question that we were interested in was, what is the size of the primary transcript of a viral RNA? At that time, it was known that cellular primary transcripts are typically much longer than their messenger RNAs—subsequently shown to result from mRNA splicing. It was not known whether there was also a precursor to the genome-length viral RNA or not, and so we generated a lot of viral cDNA from a lot of viral particles and hybridized it with radioactively pulse-labelled RNA from infected cells. We pulse-labelled as short as 5 minutes so that we could look at the primary transcript, and we determined that it is the same size as the viral genome in virions. These results now are kind of taken for granted, but at that time, that was certainly not the case.

*WB: And it laid the ground for many things that came later.*

HF: Yes, and you know, as people have understood the replication of retroviruses, it all makes sense.

Another thing that we found was that murine leukemia viruses have a glycosylated form of Gag protein. It had already been mentioned in the literature by some other researchers as tumor antigens in some murine leukemia virus-induced tumors. A graduate student, Steve Edwards, found that this glyco-Gag is an independent primary translation product of Gag, and that it is a common feature of most MuLVs. With this work, we brought glyco-Gag on the map in 1979, and we pushed it as far as we could at the time, but back then, we didn’t have the conceptual framework to fully explore it; that came later. We also did our first genetic manipulation of Moloney virus when we created a glyco-Gag mutant. That mutant could infect cells and replicate in culture; actually it replicated reasonably well, so we knew it was not absolutely required for standard viral replication. Only 20–30 years later did we get back to it and started studying it again. By now, we have come to understand that glyco-Gag is an antagonist of at least two different host restriction factors (APOBEC3 and SERINC5). Glyco-Gag still fascinates me, also because some of our experiments say that there are still other things that it does. For example, we found that glyco-Gag is important for directing virus release through lipid rafts, and also for providing structural stability to the virus particles.

We also performed interesting experiments on the pathogenesis of MuLV. We had molecularly cloned Moloney virus and by that time, it was known that the long terminal repeats are important control elements for retroviruses. In particular, the enhancer sequences in the LTR are very important for the ability of the virus to express its genes. We created a series of LTR enhancer deletions in Moloney virus and studied their effects on viral replication. At one point, we teamed up with Elwood Linney, who was working on polyomavirus (a murine DNA tumor virus) host range mutants. Normally, polyomavirus replicates in differentiated somatic cells, but the host range mutants could also replicate in undifferentiated embryonal carcinoma cells. Elwood had tracked the difference between them to enhancer changes. We tested if you could place the polyomavirus enhancer sequences into the Moloney LTR and showed that, yes, you could, and the Moloney LTR could drive expression of a reporter gene in undifferentiated EC cells if it had the polyomavirus enhancer sequences of the host range variety, while the wild-type Moloney virus LTR couldn’t.

And we built this chimeric Moloney-polyomavirus LTR back into the actual Moloney virus to see if it could replicate in undifferentiated cells and if it might induce germ cell tumors? Curiously, the virus driven by the chimeric LTR actually showed reduced leukemia in mice, i.e. the chimeric LTR did not expand its pathogenic potential but actually greatly reduced it. That led us into looking at events important for leukemogenesis. We learned that the leukemogenesis process is far more complicated than activation of cellular proto-oncogenes by insertional mutagenesis, the signature process for retroviruses that do not carry oncogenes. This chimeric virus was able to activate the same cellular proto-oncogenes that the wild-type virus activates in the less frequently occurring tumors. So that told us that the wild-type virus must be doing something more than just activating proto-oncogenes, leading to further studies where we learned that Moloney virus induces what we call a pre-leukemic state characterized by extramedullary haematopoiesis. Over the years, we investigated how this happens.

The most informative experiment can be the ones where your hypothesis is proven wrong, because it shows you that there’s more to the whole process than you first imagined.

Another exciting virus we worked on is the Jaagsiekte sheep retrovirus, which causes lung cancer in sheep. With a very talented postdoc, Massimo Palmarini, we created an infectious and oncogenic molecular clone of JSRV, which allowed us and other people in the field to do a lot of research. Probably the most noteworthy feature for us was that the envelope protein of JSRV functions as an oncogene. Working with this virus was very rewarding because of this interesting biology of the virus.

*WB: Some years after you started your lab, HIV was discovered. What did the discovery of HIV mean for you, and the field?*

HF: The retroviruses were a very hot research subject ever since the discovery of reverse transcriptase. Largely, this was in the area of cancer, since those animal retroviruses would induce tumors in experimental animals, either directly by oncogenic transformation if they carried an oncogene, or alternatively by insertional activation of cellular proto-oncogenes. Research on oncogenic retroviruses laid the foundations for modern cancer biology, most notably the discovery of cellular proto-oncogenes and their mutations in human cancer. While animal retroviruses were well recognized, what always lurked in the background was the question, is there a pathogenic human retrovirus? The first one discovered was HTLV-I, which is associated with adult T cell leukemia. But then the AIDS epidemic came upon us, and there was the big question, could AIDS be caused by a retrovirus? There were some false starts, for example that a virus closely related to HTLV is the cause of AIDS. But ultimately of course, there was the Nobel prize winning work of Montagnier and Barré-Sinoussi, who isolated HIV, or LAV as it was first called then. The race was on, once it was clear that a retrovirus, HIV, was the root cause of AIDS. A very large part of the retroviral research community pivoted to HIV, and many others came into the field, quite rightly so. The hope was that by understanding HIV replication and then developing reagents and antibodies to HIV, you could really control the epidemic. Yet, just because of the biology of HIV, that goal is still elusive, and we have not been able to develop a preventive vaccine. However, development of effective antivirals has now converted HIV/AIDS from a fatal disease into a manageable one.

HIV/AIDS contrasts with COVID, where highly effective (although not perfect) vaccines have been developed so rapidly, using new technologies such as the mRNA vaccines. The fact that researchers were able to harness these new technologies and really have a major effect on our current pandemic is amazing.

With HIV we have not been so lucky, the virus is just not that amenable to immune control.

*WB: I know that you have not transitioned into the HIV field. Was that a conscious decision?*

HF: No, I think I didn’t make a conscious decision to stay out of it, but I also didn't make a conscious decision to get into HIV in a big way. We did perform some experiments related to HIV. For instance, we generated active synthetic Tat protein.

Another area that we got into a bit was SIV pathogenesis. We had a Moloney based vector expressing beta-galactosidase which allowed us to track these replication-defective vectors in mice, and look literally at the first cells that are infected by the virus. To implement something similar, we created an SIV based vector expressing beta-galactosidase, and together with Chris Miller, we analysed which cells are the first to be infected with SIV in a macaque.

*WB: And you also focused on HIV in your teaching, in an undergraduate course specifically on HIV, and there is also a book on that topic that you published with colleagues.*

HF: Yes, that book was one we wrote shortly after we started the course (1987) because there was no textbook for our students. We have been through seven editions of that book. The focus of that book was to teach the principles of retrovirology, immunology, public health and social science aspects of AIDS in ways that a non-major could understand.

*WB: Could you elaborate on the social impact that HIV had at the time?*

HF: At the time when HIV and AIDS first appeared, it caused great fear, and the fact that it appeared in marginalized communities—gay men, injection drug users—really made it a disease that had a lot of stigma associated with it. If you were HIV infected or had AIDS, you did something wrong. And in contrast, there were people who got AIDS because of blood transfusions who were considered “innocent victims”. Historically any sexually transmitted disease does carry its stigma. You can look back to syphilis in the 1920s and 30s, when that stigma really negatively influenced the control of the epidemic. If someone doesn’t want anyone to know that they have AIDS, for fear of being avoided, then they will not let people know they have AIDS as long as they can, while remaining infectious.

The book also covers the issue of how people evaluate risks.

*WB: Did you think back at the time that scientists involved with HIV research got involved enough in educating the public about the nature of the virus, of the disease?*

HF: I think so, yes. I know when we first started teaching our course, in the early stages of the epidemic, many students who came out of the course would tell us that they were energized about the fact that they knew the real information, and that when they encountered people in their daily lives who were making false statements, they could just say “No, that’s not the case, this is what’s true.”

Providing our students with an understanding of what was really going on wasn’t just teaching them, it was actually empowering them to spread the word, to spread the facts.

*WB: That is probably quite similar to the situation in the SARS-CoV-2 pandemic. I know that my colleague who gave a lecture on SARS-CoV-2 in our lecture series provided the students with a pdf that was full of links to the appropriate sources, the correct information. Because on social media, there was so much wrong information, it was important to give the right information, the right resources, and to empower the students to spread them. Maybe it's even more of a problem today with social media, and misinformation spreading so easily.*

HF: Yes, you're right, you're completely right, it's a different world now.

*WB: Looking back over the years, to you personally, what were the most important or intriguing developments in the field of retrovirology in general?*

HF: I will mention three. One very important finding was the role of retroviruses in cancer, and particularly the role of retroviruses in the discovery of oncogenes, which really set the foundation for molecular cancer biology. That was a big one. Number two was the more recent discovery of the ability of retroviruses to counteract host restriction factors. That’s a whole field that’s obviously important for many viruses, that retroviruses have really shed light on. And the third is endogenous retroviruses. I find them utterly fascinating. These viruses provide us with archaeological footprints of viral infections over evolutionary time frames, and that’s only because they leave a DNA footprint in the genome.

*WB: Endogenous viruses are really fascinating. What I find especially intriguing is that the syncytin genes, which are so important for placenta formation, are actually endogenous retrovirus env proteins. And the finding that different mammalian species harbor different syncytins because of multiple independent events of exaptation is really fascinating. Looking at the big picture, viruses have been so crucial in evolution.*

HF: And on the evolutionary scale, it gives us an idea of how much viruses in general really shape how species develop, and we just happen to have a record of them for the retroviruses.

One of the most fascinating systems right now is the koala retrovirus, because there is ongoing germline infection and endogenization in our time scale. We get to look at what happens, and to understand the relationship of the footprints that we see of an endogenous virus relative to the scope of the infection in the species.

*WB: If you look back, what were your biggest challenges in developing your career?*

**Fig. 4:**
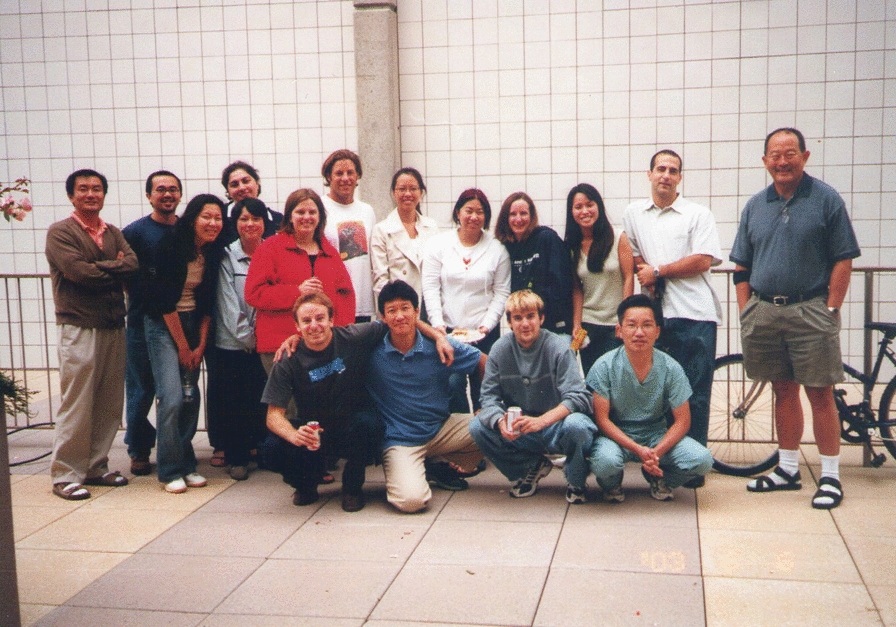
Hung Fan’s lab group, circa 2005

HF: I think for me the most challenging thing in my academic career was when I had been at the Salk Institute for six or seven years, and I didn’t get promoted. It became clear that I needed to look for another position. And you know, that is always a personal blow. Since for us scientists, our scientific success is also a measure of personal validation. So I had to look around and I found the position at UC Irvine and I moved here, and I spent the rest of my academic career here (Fig. 4).

The lesson I learned was that even if it’s painful, you have to pick yourself up and keep going. And also I realized that they had made their final decision, but I could say to myself “Well, that’s their decision, but that doesn’t necessarily mean that I don’t have any worth."

Also ultimately, the move was a really good thing. The environment at the Salk Institute was a great place to start a research program—all you did was research, and there were no other calls on your time. At the same time, while this environment was very nurturing, it could also be a little bit confining. Everybody you interacted with kind of thought along the same lines and was working on the same issues. Moving to a university environment, my colleagues, also in my department in particular, were from a considerably wider range of research outlooks and fields, and that allowed my horizons to expand.

At UCI, there was a graduate program that allowed me to build a research program with a large component of graduate students in it. This was important both because I enjoyed interacting with the students, and of course they were the hands in the lab who did a lot of the experiments.

This move also created the opportunity to become involved in academia in ways other than my research program itself. Over the years, I became the director of the UCI Cancer Research Institute, although my own field of cancer virology is only a small part of cancer research. I remained in that position for almost 30 years and during that time, with a clinical colleague, we developed the Cancer Center at UCI which is an NCI designated cancer center. I oversaw the basic science portion of the cancer effort at UCI, and that’s something that I found very rewarding.

The other thing was that in the university environment you are involved in both undergraduate and graduate teaching, which was also very important to me. For example, I started the course on HIV in 1987 for undergraduate non-majors and taught that course until I retired.

In academia, there are also many other things to get involved in, for example I served on our university faculty promotions committee for many years. That was an important function for the university but it also gave me a lot of insight into how academic quality should be evaluated.

*WB: So what is your outlook then on how academic quality should be evaluated? Maybe also in contrast to how it currently is evaluated?*

HF: Let me say one thing first. My path into research and academia was from probably two generations back, and it was much easier than for our young colleagues today. As my narrative describes, I didn’t have to go out and hunt very hard for a job, it was offered to me. Nowadays, that’s definitely not the case. In our American academic environment, our faculty have to be almost superhuman to survive. If you’re in the STEM fields, by the time you come up for tenure, if it is not entirely clear that you have a path forward for your scholarly activities, then you’re not going to succeed. In our field, that largely means you have to obtain significant research funding just to buy the supplies and pay for graduate students and postdocs. Currently, the paylines at NIH are very challenging, so our young faculty but also established faculty, spend a lot of time writing grants. While it is important, the writing of a grant in itself is not productive for research.

Then there are also other demands on faculty members, such as teaching, which is taken very seriously. In my department, teaching was quite substantial, with classroom teaching, amounting to maybe 50 h a year, and that of course is in addition to the mentoring of undergraduate and graduate students and postdocs. Then of course, a faculty member also has to serve on committees, be a part of the university community and contribute to this community. For our young faculty, that amounts to many duties and demands, and it’s quite a challenge.

I told students who were in my lab, if you want to go into academia, you really have to love the research, doing the research has to be a reward in and of itself, as opposed to getting a paycheck. There may be other avenues for our trainees where they may be able to better balance their lives. But if you’re going into academia, you have to do it because you understand the challenges and you really are committed to doing the research, teaching and service.

*WB: What do you suggest, is there a way to improve this situation?*

HF: I do know that our university is putting more attention into mentoring our young faculty, and in particular in assigning mentors to young faculty as soon as they arrive. The worst thing that can happen for a young faculty member is to start his/her career without watching the tenure clock. In our system, there are some pretty standardized times at which important decisions such as tenure are made. When someone first starts out, a tenure decision in 6 or 7 years seems like a long time away. But actually, time goes very quickly: if a tenure decision is made after six years, the review is actually taking place after 5 years, and if you then count backward, all of the activities, all of the success in research actually has to be accomplished in something closer to 4 years. That is a very tight timeline. So, the mentors can help the young faculty to keep everything on track.

Universities are also offering workshops, or as we call them boot camps, on grant writing and other things. If you’re successful at getting a grant, that’s taking a big load off, although you probably start sweating the renewal right away.

*WB: So you would say that the pressures are higher than they used to be?*

HF: I think so, yes. In our American University system, we have the tenure structure, and there’s tremendous pressure for the first few years until someone gets tenure. After that, there is less pressure. Of course, there are also many institutions that don’t have tenure, but you have the continual pressure of obtaining sufficient grant funding to carry out your research.

*WB: Yes, and that brings another sort of pressure when you need to get funding for Ph.D. students or postdocs in the lab. Even if your own position is permanent, there are other people who rely on you. For example, if you have funding for only part of a Ph.D. phase and you need to obtain a new grant half-way through the project, that is really stressful. If someone has already invested a lot of time and effort, they really rely on your ability to get more funding, and of course you don’t want to let them down.*

HF: Yes, you're right. As you build a research group, you’re really carrying both the immediate and long-term future of people on yourself, there’s no question about that.

*WB: So what is the best advice, maybe advice that you got yourself during your career, on how to balance everything? What is the best advice that you routinely passed on to the students in your lab on handling all that?*

HF: You know, I don't think I got advice on that as I was training. I really was kind of a gung ho lab rat as a graduate student and postdoc, and so were the people around me. That was kind of the culture, the environment that I had when I was at the Salk Institute. One of the benefits of being in that young lab was that we not only worked together, but we often played together, too. And so, there was a support community in that sense. When I moved to UCI, I didn’t need that kind of support anymore, I already had research grants and was building the lab fairly quickly.

Now, what kind of advice do I give students? From my experience I would say, having a peer group, someone who can relate is really important. I think my own research group was usually of a size where the students and postdocs in the lab could provide each other with this kind of support. They also played together, and that’s also part of support. I think if you have a much smaller group or it’s physically isolated, that might be more of an issue.

With postdocs, I have also certainly chatted about what their long-term plans are and where they have questions, where I could provide advice. And I have of course provided whatever advice I could tender, and that’s also continued after they’ve moved to their next positions. I’m always happy to give my perspective on a situation. One of my graduate students said that she’d enjoyed the fact that I gave her space, but I was there when she needed advice.

*WB: Besides having a peer group, are there other things that you have found helpful in staying balanced? As we discussed, there are many stressors, even if you thoroughly enjoy your work. You maybe see that double-bass standing behind me, and the saxophones back there, so for me, music is really important to keep some balance. It can be therapeutic.*

HF: Ah yes, like you, I find that music has always been a good balance for me, giving me some right brain activity. I play the violin and the viola, and I’ve played chamber music for many years. When I first got to the Salk Institute, I joined a string quartet and we played together the whole time I was there. When I came here to the UCI, I also found friends to play music with and we all try to get together once a week. And you’re right, it provides you another release. Also, the enjoyment in playing music is not only the music itself, but it’s actually communicating with other musicians in a different medium. That’s a lot of fun, and I enjoy it.

There was a legendary scientist at the Salk Institute named Marguerite Vogt. Marguerite Vogt was someone who was originally from Germany, she had first started as a Drosophila geneticist and did pioneering work in that field. She came to the Salk Institute and worked in the laboratory of Renato Dulbecco, and she was the hands that did some of the most important experiments that they did as a team, for which she wasn’t recognized as much as she should have been. But anyway, Marguerite was not married and she lived by herself, and she would spend 6 days a week in the lab from seven in the morning until five in the evening. She was very intense and always thinking and very excited about science. But Sunday was her day off. And every Sunday, Marguerite’s house was open for music. She had a very nice piano in her home and a good music library, and starting at 10 o’clock, people would come to her house and play music. At 12:30, a wine and cheese lunch would be served, and conversations would turn around anything but science. That was an unspoken rule, that you did not talk about science during those hours as well.

*WB: Ah yes, that does sound lovely. We have talked about some problems you see in academia today. Would you say there have also been positive changes?*

HF: I would say yes. When I started, there were very few women in the upper echelons of science, certainly in the upper echelons of biology. That is something that has changed over the years, and particularly over the last 15 years. The community has recognized that as an important issue that needs to be continually addressed, and I think that is a very positive thing. We need to recognize that women and people of different ethnic minorities have an additional burden that they have to work against. 50 years ago, there was very overt discrimination against women, against certain ethnic groups. Even today, there still is, but it is officially not acceptable. While there is a shift in our culture that bias is not acceptable, there still are underlying assumptions, presumptions that that need to be worked against continually. Its positive part is that bias is getting recognized, but it is still a work in progress.

*WB: We probably need to remember that and not get complacent. Even if some things seem perfectly normal to us, such as interacting naturally with many people from abroad in the lab, that is not necessarily their experience in their day-to-day life.*

HF: Yes, I think the good thing about academia is that the primary criterion is excellence, and I think excellence, by and large, is becoming color blind and gender blind. Whoever publishes excellent work is going to be recognized.

We have made some progress in that more than half of the graduate students in biology are female. We’re not doing as well in terms of African-Americans and Hispanics, in the US at least.

While excellence really is the coin of the realm as people ascend in the ranks, is there equal access and reward for quality across genders and ethnic groups? It’s good that we have recognized the problem, and the challenge is to find solutions.

*WB: Have you ever had any bad experiences yourself, being Chinese? Did you ever have the impression that this played a role at all in how people perceived you?*

HF: I don't think so. I’m in many ways an outlier, and I’d contrast myself to someone who was born and raised in China and came to the US as an adult. At the time I was growing up, there were many fewer Asians in the US and in science than there are now. There’s this funny thing that happens: if you’re a very low percentage minority, you’re almost not considered a minority. It’s when numbers begin to grow that people begin to worry about, what is the effect of this wave of people coming from another place. Might they be taking our jobs?

I was raised speaking English and I speak English with a pretty flat American accent, and probably people would not classify me as a Chinese immigrant as much as a Chinese American. I did not perceive racial prejudice as a barrier to my success in academia. Certainly, it could have occurred but I wasn’t aware of it. At least I never felt that I should have gotten something that I didn’t get, and the reason that I didn’t get it was because I’m Asian. Or gay.

*WB: And would you say it also never played a role in your daily life, or at least you didn’t perceive it as playing a role?*

HF: I did not perceive it as playing a role. Again, this could be because of where I've lived. It's just not been an issue.

*WB: And you have been to China on a few occasions if I remember correctly. Do you speak Chinese; I believe your parents spoke English at home.*

**Fig. 5:**
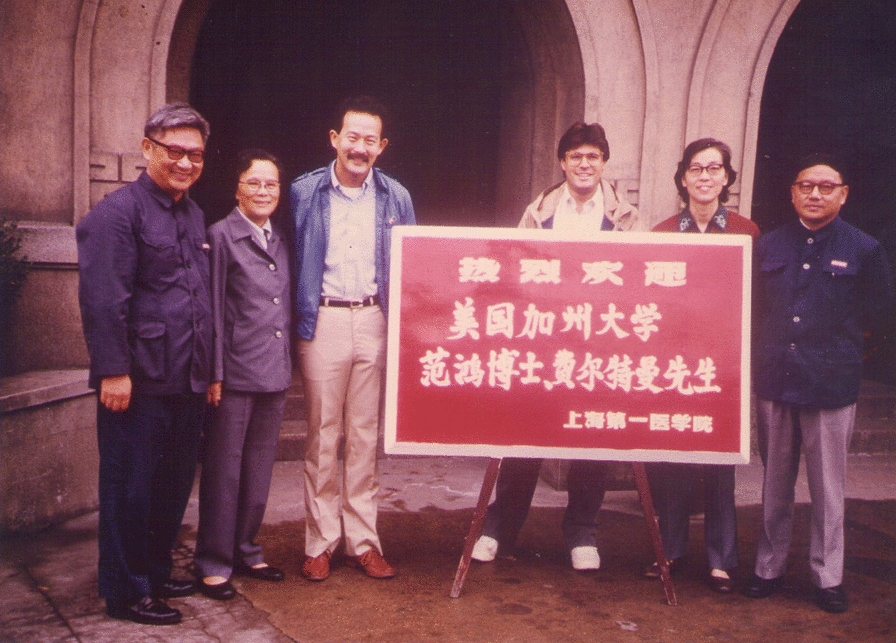
Hung Fan’s first visit to the First Shanghai Medical College (now Fudan University Medical School). The woman to his right was Prof. Zheng Bao-fen, his host

HF: Yes, that's true. The first time I came to China was in 1984, which was pretty early for western travelers (Fig. 5); in those days, everybody was riding bicycles, and wore Mao jackets … It was very illuminating, because I found that I could resonate with the people there, because we still had some common outlooks. Even though I was raised in the US, in Indiana, my parents still had many of the Chinese outlooks, and that was something I recognized.

I do understand a little bit of Chinese, with two provisions: I have very limited vocabulary, and I can most easily understand people who are older, like the Chinese I heard when my parents spoke Chinese around us—although they mostly spoke English to us.

And when I go to China, I am recognized as an American very quickly. There is more than facial appearance, it’s also how you carry yourself.

*WB: Oh, that’s funny, I wouldn’t have guessed it’s so easy to recognize. Have you ever felt when you grew up that you were missing something, some part of your culture?*

HF: No, I think anybody who grows up deals with feeling different, right? That feeling different might be focused on many things, and one obvious thing for me when I was growing up was that I looked different from everybody else, I did not look like the rest of my classmates in high school. But that feeling of being different was probably not that uncommon among my classmates as well, they just may have focused on something different that made them feel different.

*WB: Maybe at the end let me ask, what would you like to tell young people in retrovirology?*

HF: I guess the major thing I would tell a young person in our field is, there are various reasons to do it, and there are no right or wrong reasons. For some people doing retrovirology, or biology in general, may be the way to a promising future in biotechnology, or into business. There’s nothing intrinsically more or less valuable in that compared to someone who is in retrovirology for the love of doing experiments, who just wants to be a lab tech, who does not care or worry about getting grants. And then there are those who want to be traditional academicians, who want to do the whole works. The most important thing is to be committed and to enjoy the research. If you don’t enjoy it, it’s just going to be very hard. But if you enjoy it, and if it seizes your imagination, that imagination and commitment will take you through a lot of nights and days when things seem really tough.

*WB: That message is a perfect ending to our conversation. Thank you so much, Hung, for taking the time and sharing your insights.*

